# SHU00238 Promotes Colorectal Cancer Cell Apoptosis Through miR-4701-3p and miR-4793-3p

**DOI:** 10.3389/fgene.2019.01320

**Published:** 2020-01-10

**Authors:** Haoyu Wang, Yurui Ma, Yifan Lin, Rui Chen, Bin Xu, Jiali Deng

**Affiliations:** ^1^ Department of Chemistry, Qianweichang College, Shanghai University, Shanghai, China; ^2^ School of Life Science, Shanghai University, Shanghai, China; ^3^ Innovative Drug Research Center, Shanghai University, Shanghai, China

**Keywords:** SHU00238, colorectal cancer, miR-4701-3p, miR-4793-3p, target

## Abstract

Colorectal cancer is one of the most leading causes of death. Searching for new therapeutic targets for colorectal cancer is urgently needed. SHU00238, an isoxazole derivative, was reported to suppress colorectal tumor growth through microRNAs. But the underlying mechanisms still remain unknown. Here, we explored the mechanism of SHU00238 on colorectal cancer by RT-PCR, CCK-8, flow cytometry, mirTarBase, and GO enrichment analysis. We screened partial microRNAs regulated by SHU00238 in colorectal cancer cells. Furthermore, we identified that miR-4701-3p and miR-4793-3p can reverse the acceleration of SHU00238 on colorectal cancer cell apoptosis in HCT116 Cells. Finally, we found that SMARCA5, MBD3, VPS53, EHD4 are estimated to mediate the regulation of miR-4701-3p and miR-4793-3p on colorectal cancer cell apoptosis, which targets ATP-dependent chromatin remodeling pathway and endocytic recycling pathway. Taken together, our study reveals that SHU00238 promotes colorectal cancer cell apoptosis through miR-4701-3p and miR-4793-3p, which provide a potential drug target and therapeutic strategy for colorectal cancer.

## Introduction

Colorectal cancer (CRC) is one of the most common malignant tumors that endanger human health. According to the latest statistics, the incidence of CRC ranks third among malignant tumors worldwide and the mortality rate ranks second. It is estimated to be more than 1.8 million new cases and 881,000 deaths in 2018 (Saus et al., 2016; Bray et al., 2018). However, the pathogenesis of CRC is not clear and the prognosis is poor. At present, the treatment of CRC mainly uses surgery, radiotherapy, chemotherapy, exercises, and chemical synthetic antineoplastic drugs (Nilsson et al., 2013; Kuipers et al., 2015; Mach and Fuster-Botella, 2017; Batacan et al., 2018). However, most of the patients had been diagnosed in the middle and late stages and were insensitive to radiotherapy (Hosseinimehr et al., 2019), and new antineoplastic drugs are expensive and have some side effects (Baker et al., 2000). Therefore, searching for highly effective and low toxic antineoplastic drugs is the focus of current treatment for CRC.

SHU00238 is an isoxazole derivative, which was reported to suppress colorectal tumor growth through microRNAs (Wang et al., 2019). But the underlying mechanisms still remain unknown. MicroRNAs are noncoding single strand RNAs (Zhou et al., 2019), encoded by endogenous genes with a length of about 18–25 nucleotides (Wang et al., 2018), which play a vital role in the inhibition of posttranscriptional translation of mRNAs (Ambros, 2004; Bartel, 2004). Two microRNAs from the opposite side of the pre-microRNA name -3p or -5p (Voinnet, 2009). In recent years, studies have discovered that microRNAs play an essential role in the pathogenesis of CRC. Dysregulated patterns of expression of microRNAs such as miR-320a and miR-21 are associated with CRC cell proliferation and migration (Sun et al., 2012; Xiong et al., 2013), while miR-34a and miR-365 are implicated in the control of CRC cell apoptosis (Yamakuchi et al., 2008; Nie et al., 2012; Liu et al., 2019). These microRNAs have been reported to regulate target genes and contribute to CRC.

In this present study, we first screened partial microRNAs regulated by SHU00238 in CRC cells. Furthermore, we identified that miR-4701-3p and miR-4793-3p can reverse the acceleration of SHU00238 on CRC cell apoptosis in HCT116 Cells by CCK-8 and flow cytometry. Finally, we used miRNA target prediction algorithms and GO enrichment analysis and revealed that SMARCA5, MBD3, VPS53, and EHD4 are estimated to mediate the regulation of miR-4701-3p and miR-4793-3p on CRC cell apoptosis, which targets ATP-dependent chromatin remodeling pathway and endocytic recycling pathway. Taken together, our study reveals that SHU00238 promotes CRC cell apoptosis through miR-4701-3p and miR-4793-3p, which provide a potential drug target and therapeutic strategy for CRC.

## Materials and Methods

### Cell Culture

Human CRC cell HCT116 was purchased from Chinese Academy of Sciences (CAS) Cell Bank (Shanghai, China). Cells were cultured in Dulbecco’s modified Eagle medium (DMEM, Corning, NY, USA) supplemented with 10% fetal bovine serum (FBS, CellMax, Shanghai, China) and 1% penicillin-streptomycin (PS, Thermo Scientific, MA, USA) at 37°C in a 5% CO2 atmosphere.

### Transfection and Treatment

HCT116 cells were transfected with microRNAs mimics (50 nM; RiboBio, Guangzhou, China), inhibitors (75 nM; RiboBio, Guangzhou, China), or negative control for 8 h using Lipofectamine 2000 (Invitrogen, CA, USA) as manufacturer’s instructions, followed by SHU00238 (0.3 μM) or DMSO treatment for 48 h. The microRNAs mimics, inhibitors and negative control were purchased from RiboBio (Guangzhou, China).

### Cell Viability Assay

Cell viability was detected with cell counting kit-8 (CCK-8, Bioworld, Shanghai, China). HCT116 cells were seeded in 96-well culture plate at 2 × 104 cells/well and treated as indicated. Cells were incubated with CCK-8 at 37°C for 1 h. The optical density was measured at 450 nm by microplate reader (Bio-Rad, CA, USA).

### Cell Apoptosis Assay

Annexin V-FITC Apoptosis Detection Kit (Beyotime, Shanghai, China) was used to measure the apoptosis level of HCT116 cells treated as indicated. Cells were stained with Annexin V and PI according to the instructions and then analyzed by flow cytometry (Beckman, CA, USA).

### Real-Time PCR

RNA isolation and relative quantification RT-PCR were performed as described previously (Xiao et al., 2017; Deng et al., 2019). The microRNAs primers were purchased from RiboBio (Guangzhou, China).

### Analysis of miRNA Target Genes and Possible Downstream Signaling Pathway

The downstream target genes of a miRNA were screened using the miRTarBase website (http://mirtarbase.mbc.nctu.edu.tw/php/index.php), and the common downstream target genes of miRNAs were obtained by comparison. DAVID website (https://david.ncifcrf.gov/home.jsp) was used for GO ONTOLOGY and PATHWAY analysis to screen metabolic pathways and gene products significant affected by common target genes.

### Statistical Analysis

All data were presented as mean ± SEM from three independent experiments. All statistical analyses were performed through IBM SPSS Statistics 20 (Armonk, NY, USA). Comparison of quantitative data was performed using an independent-samples t-test or one-way ANOVA test followed by Bonferroni’s *post hoc* test. P Values < 0.05 were considered statistically significant.

## Results

### SHU00238 Regulates Partial miRNAs in CRC Cells

**Figure 1:**
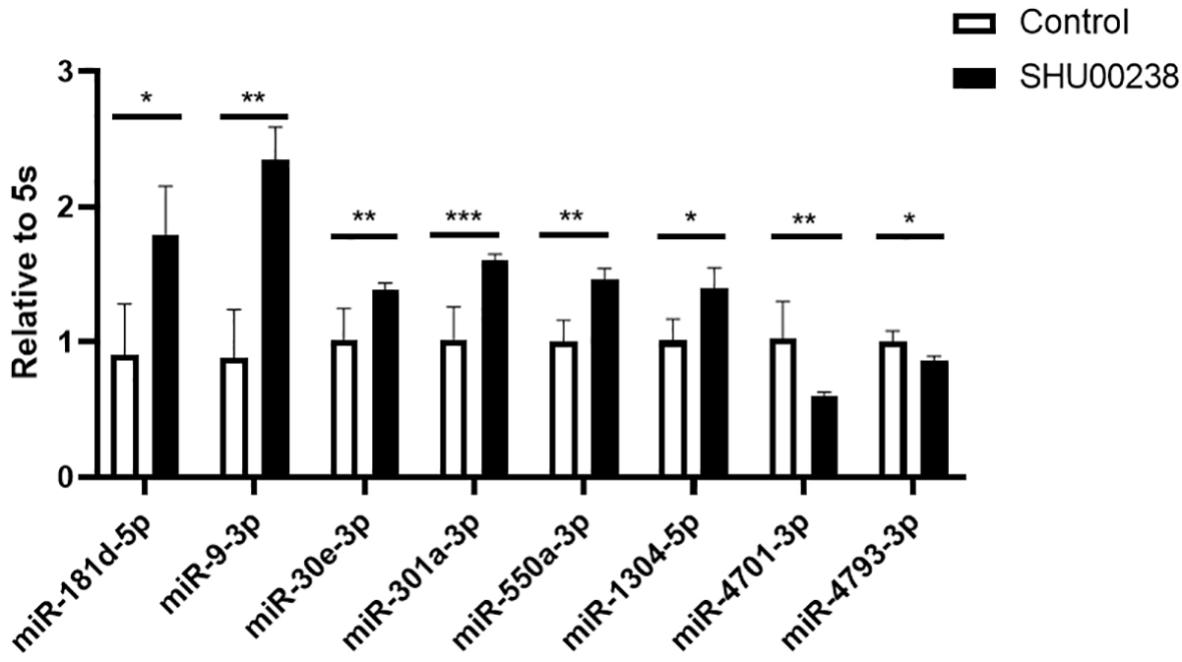
SHU00238 regulates partial miRNAs in colorectal cancer cells. All values were the average of at least three biological replicates, and the data shown are mean ± SEM. *P < 0.05, **P < 0.01, ***P < 0.001.

Preliminary data showed that SHU00238 could regulate a set of miRNAs (Wang et al., 2019). Here, we examined the mRNA level of these miRNAs in HCT116 cells treated with SHU00238 or DMSO. As shown in **Figure 1**, miR-181d-5p, miR-9-3p, miR-30e-3p, mir-550a-3p, and miR-1304-5p are upregulated, while miR-4701-3p and miR-4793-3p are downregulated by SHU00238.

### MiR-4701-3p and MiR-4793-3p Reverse the Acceleration of SHU00238 on CRC Cell Apoptosis in HCT116 Cells

**Figure 2:**
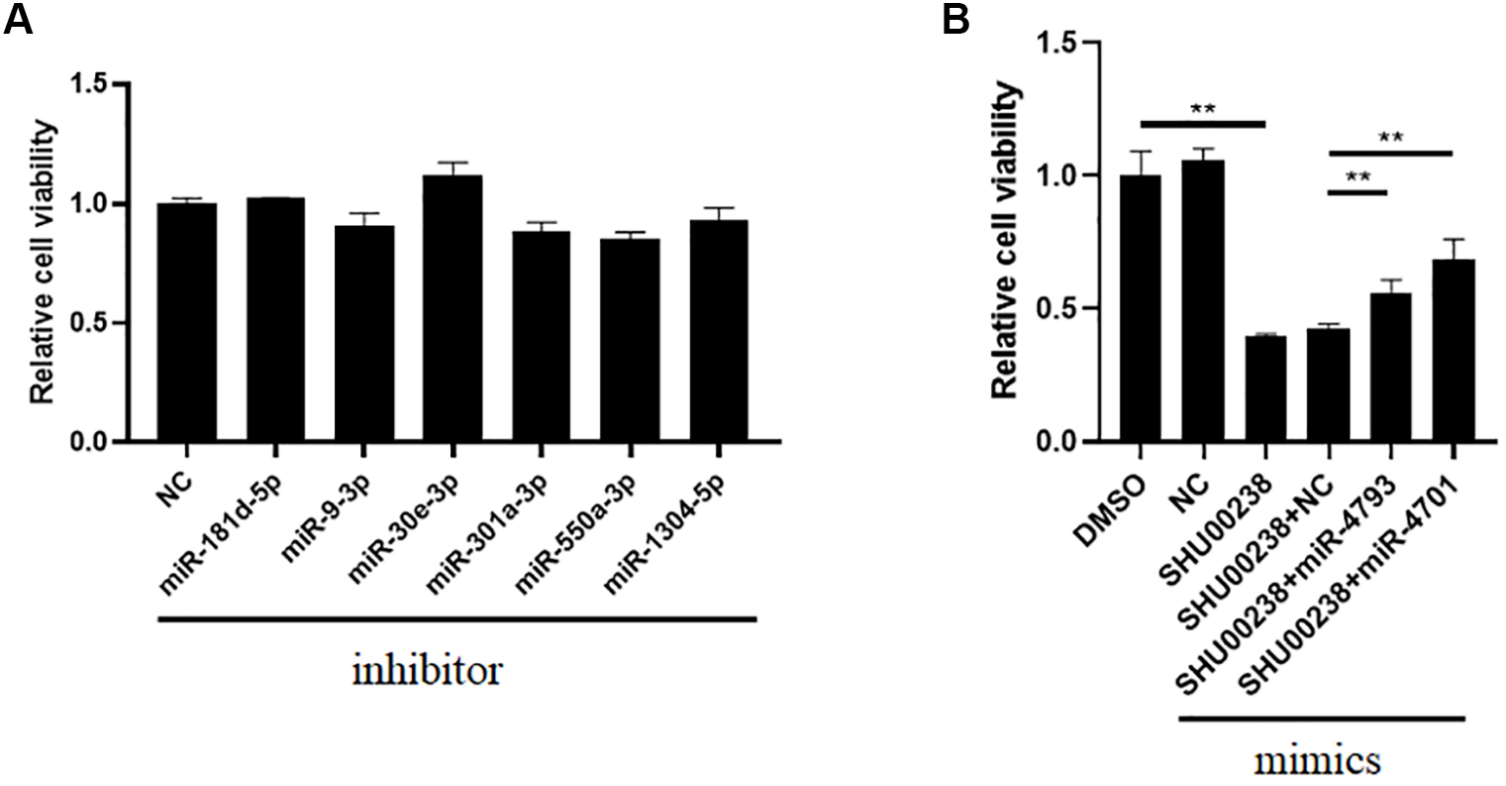
MiR-4701-3p and miR-4793-3p reverse the inhibition of SHU00238 on HCT116 cell viability. **(A)** Cell viability analysis of HT116 cells transfected with miR-181d-5p, miR-9-3p, miR-30e-3p, mir-550a-3p, miR-1304-5p inhibitors or NC, followed by SHU00238 treatment, was analyzed by CCK-8 kit. **(B)** Cell viability analysis of HT116 cells transfected with miR-4701-3p and miR-4793-3p mimics or NC, followed by SHU00238 treatment, was analyzed by CCK-8 assay. All values were the average of at least three biological replicates, and the data shown are mean ± SEM. **P < 0.01 relative to the control.

**Figure 3:**
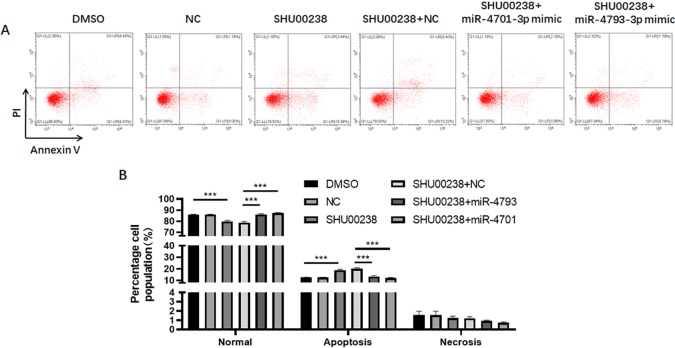
MiR-4701-3p and miR-4793-3p reverse the acceleration of SHU00238 on colorectal cancer cell apoptosis in HCT116 Cells. **(A)** Apoptosis level of HCT116 cells transfected with miR-4701-3p and miR-4793-3p mimics or NC, followed by SHU00238 treatment, was measured by Annexin V-FITC Apoptosis Detection Kit. **(B)** The Statistical results of apoptosis and necrosis analysis of HCT116 cells transfected with miR-4701-3p and miR-4793-3p mimics or NC, followed by SHU00238 treatment. All values were the average of at least three biological replicates, and the data shown are mean ± SEM. ***P < 0.001 relative to the control.

To investigate the downstream of SHU00238 on CRC cell apoptosis, we examined the reverse ability of miR-181d-5p, miR-9-3p, miR-30e-3p, mir-550a-3p, miR-1304-5p, miR-4701-3p, and miR-4793-3p in HCT116 cells treated with SHU00238 with CCK-8 assay. The results showed that miR-181d-5p, miR-9-3p, miR-30e-3p, mir-550a-3p, and miR-1304-5p cannot reverse the inhibition of SHU00238 on HCT116 cell viability (**Figure 2A**), while miR-4701-3p and miR-4793-3p reverse the inhibition of SHU00238 on HCT116 cell viability (**Figure 2B**). Furthermore, we detected the cell apoptosis and necrosis by Annexin V and PI staining, we found that miR-4701-3p and miR-4793-3p` reverse the acceleration of SHU00238 on CRC cell apoptosis in HCT116 Cells (**Figure 3**).

### SMARCA5, MBD3, VPS53, EHD4 Are Estimated to Mediate the Regulation of miR-4701-3p and miR-4793-3p on CRC Cell Apoptosis

**Figure 4:**
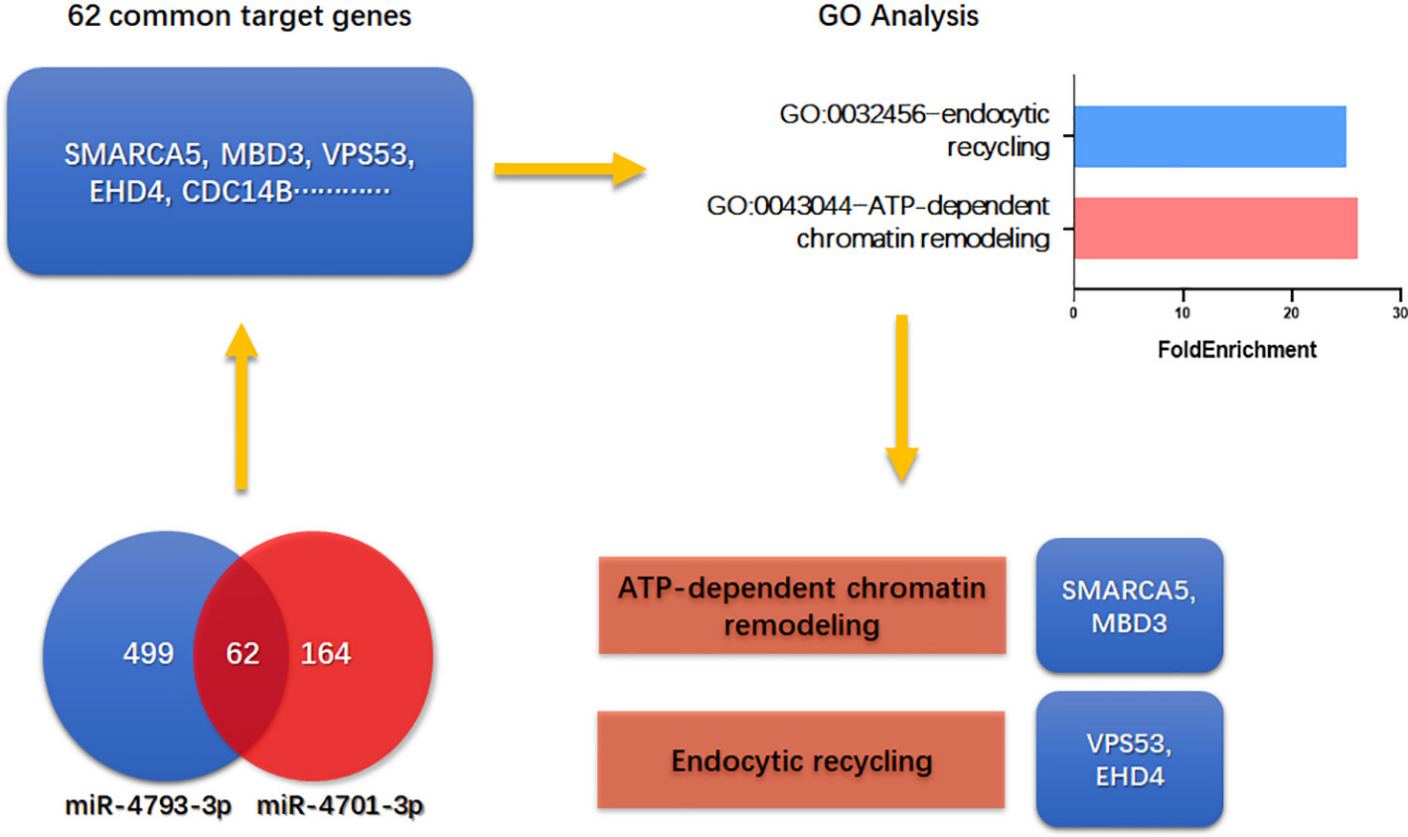
SMARCA5, MBD3, VPS53, and EHD4 are estimated to mediate the regulation of miR-4701-3p and miR-4793-3p on colorectal cancer cell apoptosis.

To investigate the underlying mechanism of miR-4701-3p and miR-4793-3p on CRC cell apoptosis, we screened 62 common targets of miR-4701-3p and miR-4793-3p through mirTarBase including SMARCA5, MBD3, VPS53, EHD4, and so on. GO enrichment analysis revealed that ATP-dependent chromatin remodeling pathway and endocytic recycling pathway were significantly changed by the targets of miR-4701-3p or miR-4793-3p. SMARCA5 and MBD3 are associated with ATP-dependent chromatin remodeling (Aydin et al., 2014; Biswas et al., 2019), VPS53 and EHD4 are related to endocytic recycling (George et al., 2011; Feinstein et al., 2014) (**Figure 4**). Overall, our study demonstrates that SHU00238 promotes CRC cell apoptosis through miR-4701-3p and miR-4793-3p.

## Discussion

Isoxazole derivatives play important roles in antitumor (Jensen et al., 2008; Zhu et al., 2018). Our previous data showed SHU00238, an isoxazole derivative, suppresses colorectal tumor growth through microRNAs (Wang et al., 2019). In this study, we screened partial microRNAs regulated by SHU00238 in CRC cells. Further analysis showed that miR-4701-3p and miR-4793-3p can reverse the acceleration of SHU00238 on CRC cell apoptosis in HCT116 Cells.

Taken together, our study reveals that SHU00238 promotes CRC cell apoptosis through miR-4701-3p and miR-4793-3p, which provide a potential drug target and therapeutic strategy for CRC. However, extensive studies are needed to explore the participation of miR-4701-3p and miR-4793-3p in the regulation of SHU00238 on CRC tumor in animals and human (Liang et al., 2017).

MiR-4701-3p, derived from exosomes, was shown to influence clopidogrel-induced liver injury, platelet reactivity, and drug-induced toxicity (Freitas et al., 2016; de Freitas et al., 2017). MiR-4701-3p was downregulated in patients with high risk of cadiovascular disease. BTNL3 and CFD mRNAs are regulated by miR-4701-3p. BTNL3 is involved in proliferation, development, inflammation, and immune response. CFD has a role in coronary heart disease. (Freitas et al., 2016). But miR-4701-3p has not been identified in cancer especially in CRC. The research showed that miR-4793-3p might be associated with angiogenesis, arginine metabolism, cell adhesion and chemotaxis, extracellular matrix remodeling, hypoxia/oxidative stress, inflammation, and muscle contraction (Ng et al., 2015). MiR-4793-3p is significantly increased in small bowel tissues of necrotizing enterocolitis compared with control tissues. TLR4 is a target of miR-4793-3p. Recent study identified miR-4793-3p was differently express in hepatocellular carcinoma (Pascut et al., 2019). More and more research showed that abundant microRNAs play a vital role in CRC. For example, miR-375 inhibits CRC cell proliferation mainly through targeting both JAK2/STAT3 and MAP3K8/ERK signaling pathways (Wei et al., 2017), miR-30a regulates cell proliferation and tumor growth of CRC by targeting CD73 (Xie et al., 2017), miR-374b regulates CRC cell apoptosis (Gong et al., 2017). But the role of miR-4701-3p and miR-4793-3p which we identified in this study in CRC cell proliferation and apoptosis remains unknown.

In addition, miRNA target prediction algorithms and GO enrichment analysis revealed that SMARCA5, MBD3, VPS53, and EHD4 may mediate the regulation of miR-4701-3p and miR-4793-3p on CRC cell apoptosis, which targets ATP-dependent chromatin remodeling pathway and endocytic recycling pathway. Gut-specific conditional deletion of mbd3 mice showed a large increase in proliferating cells in the colon after DSS treatment compared to control animals, markedly increased susceptibility to colitis-induced tumorigenesis *via* c-Jun-MBD5/NuRD-AP-1 signaling. Recent study showed that MBD3 is a target of miR-8073, which is reported to be a colorectal tumor suppressor (Mizoguchi et al., 2018). There are no report about the role of SMARCA5, VPS53, and EHD4 in CRC. Further studies are needed to explore whether SMARCA5, MBD3, VPS53, EHD4 mediate miR-4701-3p, and miR-4793-3p’s function *in vivo* and *in vitro* (Jiao et al., 2017).

Chromatin remodeling means position or composition of a nucleosome is altered in the chromatin, including ATP-dependent and ATP-independent pathway. ATP-dependent chromatin remodeling pathway constitutes the majority of the remodeling activity (Mayes et al., 2014). The important member of ATP-dependent chromatin remodeling pathway is SWI/SNF, which contains a Swi2/Snf2 ATPase subunit (Osley et al., 2007). ATP-dependent chromatin remodeling pathway participates in DNA damage repair (van Attikum and Gasser, 2005), coronary development (Huang et al., 2008), pancreatic neuroendocrine tumors (Clynes et al., 2013), epithelioid sarcomas (Kohashi and Oda, 2017). But the participation of ATP-dependent chromatin remodeling pathway in CRC remains unknown. Cells transport extracellular materials into cells by endocytosis. The endocytic recycling pathway returns most of the protein and lipids to the plasma membrane. The balance between endocytosis and recycling controls the composition of plasma membrane and participates in many processes (Grant and Donaldson, 2009). These recycling pathways are essential for maintaining the proper composition of various organelles and for returning essential molecules that carry out specific functions to the appropriate compartments (Maxfield and McGraw, 2004). Endocytic recycling pathway plays roles in cell adhesion, morphogenesis, cell fusion, learning, and memory (Grant and Donaldson, 2009), autophagy (Nixon, 2013), nutrient absorption, immune response (Lin and Shi, 2019), CRC metastasis (Huang et al., 2019). Further studies are needed to explore whether endocytic recycling pathway mediate CRC cell proliferation and apoptosis.

In summary, we identified miR-4701-3p and miR-4793-3p mediate SHU00238’s effect on CRC cell apoptosis, which open a new approach for CRC.

## Data Availability Statement

The data for this study are available by contacting the corresponding author.

## Author Contributions

HW and YM carried out the experiments. YL, RC, and BX contributed to the helpful discussion. JD directed the project, wrote and edited the manuscript. All authors read and approved the manuscript.

## Funding

This work was supported by the National Natural Science Foundation of China [81700761 to JD], China Postdoctoral Science Foundation [2016M601664 to JD], and Innovation Program of Shanghai Municipal Education Commission (No. 2019-01-07-00-09-E00008 to BX).

## Conflict of Interest

The authors declare that the research was conducted in the absence of any commercial or financial relationships that could be construed as a potential conflict of interest.
